# Eye-Strain in Health and Disease

**Published:** 1901-03

**Authors:** 


**Eye-Strain in Health and Disease.** By AMBROSE L. RANNEY, M.D. Pp. viii., 321. Philadelphia: The F. A. Davis Co. 1897.

It is a well-recognised fact, not only among ophthalmic surgeons, but also among members of the medical profession in general, that the various symptoms grouped under the name of asthenopia, and many headaches, are caused by errors of refraction, or less frequently by some irregularity in the balance of the muscles which control the co-ordinated movements of the eyes. Dr. Ranney, however, as a neurologist, goes much further than this, and considers that all kinds of diseases may have faulty ocular conditions as their exciting cause. Eye-strain, in his opinion, may set up such widely different and serious nervous affections as sleeplessness, prostration, insanity, epilepsy, chorea, and even chronic gastric and digestive disturbances.

The book contains a description of the usual methods of testing the eyesight, and gives a clear description of how to examine the muscle balance and its abnormalities. The author quotes in it numerous cases in which he claims to have relieved or cured patients suffering from the various diseases enumerated above by treatment of their ametropia (focal errors) by lenses, and more particularly of their heterophoria (faulty muscle balance) by prisms or by operation on the abnormal muscles. Many of these experiences border on the miraculous.

